# Inflammatory interferon activates HIF-1α-mediated epithelial-to-mesenchymal transition via PI3K/AKT/mTOR pathway

**DOI:** 10.1186/s13046-018-0730-6

**Published:** 2018-03-27

**Authors:** Yen-Hsiu Yeh, Ho-Fu Hsiao, Yen-Cheng Yeh, Tien-Wen Chen, Tsai-Kun Li

**Affiliations:** 1 Department and Graduate Institute of Microbiology, College of Medicine, Taipei, Taiwan, Republic of China; 2 Center for Biotechnology, National Taiwan University, Taipei, Taiwan, Republic of China; 3 Center for Genomic Medicine, National Taiwan University, Taipei, Taiwan, Republic of China; 4 Department of Emergency Medicine, Sijhih Cathay General Hospital, New Taipei City, Taiwan, Republic of China; 5 Department of Internal Medicine, Kaohsiung Armed Forces General Hospital, Kaohsiung, Taiwan, Republic of China

**Keywords:** Tumor microenvironment, Inflammatory hypoxia, Interferon, HIF-1α, Epithelial-to-mesenchymal transition, Oncogenesis

## Abstract

**Background:**

Tumor microenvironments (TMEs) activate various axes/pathways, predominantly inflammatory and hypoxic responses, impact tumorigenesis, metastasis and therapeutic resistance significantly. Although molecular pathways of individual TME are extensively studied, evidence showing interaction and crosstalk between hypoxia and inflammation remain unclear. Thus, we examined whether interferon (IFN) could modulate both inflammatory and hypoxic responses under normoxia and its relation with cancer development.

**Methods:**

IFN was used to induce inflammation response and HIF-1α expression in various cancer cell lines. Corresponding signaling pathways were then analyzed by a combination of pharmacological inhibitors, immunoblotting, GST-Raf pull-down assays, dominant-negative and short-hairpin RNA-mediated knockdown approaches. Specifically, roles of functional HIF-1α in the IFN-induced epithelial-mesenchymal transition (EMT) and other tumorigenic propensities were examined by knockdown, pharmacological inhibition, luciferase reporter, clonogenic, anchorage-independent growth, wound-healing, vasculogenic mimicry, invasion and sphere-formation assays as well as cellular morphology observation.

**Results:**

We showed for the first time that IFN induced functional HIF-1α expression in a time- and dose- dependent manner in various cancer cell lines under both hypoxic and normoxic conditions, and then leading to an activated HIF-1α pathway in an IFN-mediated pro-inflammatory TME. IFN regulates anti-apoptosis activity, cellular metastasis, EMT and vasculogenic mimicry by a novel mechanism through mainly the activation of PI3K/AKT/mTOR axis. Subsequently, pharmacological and genetic modulations of HIF-1α, JAK, PI3K/AKT/mTOR or p38 pathways efficiently abrogate above IFN-induced tumorigenic propensities. Moreover, HIF-1α is required for the IFN-induced invasiveness, tumorigenesis and vasculogenic mimicry. Further supports for the HIF-1α-dependent tumorigenesis were obtained from results of xenograft mouse model and sphere-formation assay.

**Conclusions:**

Our mechanistic study showed an induction of HIF-1α and EMT ability in an IFN-mediated inflammatory TME and thus demonstrating a novel interaction between inflammatory and hypoxic TMEs. Moreover, targeting HIF-1α may be a potential target for inhibiting tumor tumorigenesis and EMT by decreasing cancer cells wound healing and anchorage-independent colony growth. Our results also lead to rationale guidance for developing new therapeutic strategies to prevent relapse via targeting TME-providing IFN signaling and HIF-1α programming.

**Electronic supplementary material:**

The online version of this article (10.1186/s13046-018-0730-6) contains supplementary material, which is available to authorized users.

## Background

Tumor is a complex heterogeneous biomass containing cancer cells and infiltrated leukocytes as well as extracellular matrices and surrounding stromal cells, collectively regarded as “tumor microenvironment (TME)” [1–6]. Notably, cancer cells often expose to various TMEs composed of different host cells (e.g. macrophages and fibroblasts) and secreted soluble factors (e.g. interferon, cytokines and BMP), where these TMEs play critical roles in various developmental stages as well as therapeutic response of cancer [7–11]. These relations are clearly illustrated by that the initiation and maintenance of tumors dependent largely on their ability to adapt to TME changes [12–15]. For example, epithelial-to-mesenchymal transition (EMT) that is highly regulated by cellular niches convert epithelial cells into migratory/invasive mesenchymal cells and TME-induced pathological EMT also contributes cancer progress, especially metastasis. Moreover, the fact that paracrine interactions within TMEs also greatly impact on tumorigenesis suggests TMEs not only bi-directionally communicate with tumor cells but crosstalk to each other [4, 5, 16–18]. Furthermore, it has been shown that adaptive responses in glucose metabolism contribute to macrophage migratory capacity during hypoxic condition and the later acquired therapeutic resistance of tumor cells depends largely on their ability to interact with TMEs [5, 6, 8, 19, 20]. Therefore, TMEs might present as novel anticancer targets in addition to oncogenic alterations in tumors.

Perhaps the most direct evidence that TME affects cancer development is that patients suffered from chronically inflamed tissues generally exhibit a high cancer incidence [21, 22]. Patients with ulcerative colitis (UC), one form of inflammatory bowel diseases (IBD), develop colon cancer with a 20-fold overall risk in comparison with population controls [23]. Chronic stimulation of innate immunity is known to promote inflammation-driven tumorigenesis and induce cancer progression. Through releasing a variety of cytokines, chemokines, and cytotoxic mediators such as reactive oxygen species, metalloproteinases, interleukins (ILs), and interferon (IFN), these immune components are considered key pathological inflammatory factors promoting tumor progression [22, 24, 25]. In addition, hypoxia is also a key feature of microenvironments in inflammatory conditions, for example, arthritis [17, 26] and solid tumors often situate in a hypoxic inflammatory TME due to the rapid growth [27–30]. Notably, both hypoxic and inflammatory responses could have pro- and/or anti-tumorigenic influences on oncogenic development, allowing cells to adjust the optimal cellular context via “outside-in” signaling with regulated transcription programming directed by master factors like hypoxia-inducing factors (HIFs, mainly HIF-1α), signal transducers, activators of transcription family (STAT, such as STAT1) and NF-κB [21, 22]. These two TMEs communicate to each other as well as oncogenic programs as evidenced by (i) interactions of HIF-1α-directed hypoxic responses with the NF-κB-mediated inflammatory programs [7, 31, 32]; (ii) HIF-1α directs tumorigenic EMT; and (iii) HIF-1α interacts with oncogenic factors such as Myc and Ras oncogenes [6, 11, 33–38]. Though molecular mechanisms underlying the crosstalk of TMEs with oncogenic programs remain largely unclear, modulations of hypoxic inflammatory responses have been seriously considered as an attractive novel target for cancer therapy [29, 39].

Under hypoxia, HIF-1α protein is stabilized and its activity is increased mainly due to blockage of hydroxylation and proteasome degradation, followed by activation of a defined set of transcriptional programming. This set of target genes encodes proteins functioning in cellular processes such as erythropoiesis, glycolysis, EMT, metastasis, angiogenesis, therapy resistance and poor prognosis [29, 39–43]. Similar to HIF-1α, NF-κB is a master transcription factor (TF) inducing expression of diverse target gene sets, thus playing an instrumental role in immune, stress and pathological inflammatory TME responses [44]. Despite of the fact that NF-κB has identified as a redox-sensitive TF, it also binds to the promoter of the *HIF-1α* gene and mediates HIF-1α expression under IL-1β, INF-γ, TNF-α and other cytokine treatments in normoxia [45–48], providing a hint that inflammatory and hypoxic transcription programs are linked. In addition, the master TF STAT3 not only mediates inflammatory IFN response and regulates expression of AKT but also involves in the growth signal-induced HIF-1α expression [49].

Cytokines such as IFN-α, through receptor interactions and subsequent induction of IFN-stimulated genes (ISGs) expression, play critical roles in inflammation [36]. IFN-α signaling pathways include the classical JAK–STAT and other auxiliary pathways such as the PI3K/mTOR/AKT and MAPK-P38 axes, and dysfunction in signaling of PI3K/PTEN/AKT/mTOR, Wnt/GSK-3 and/or Ras/Raf/MEK/ERK axes is associated tightly with cancer progression and therapeutic resistance [50]. Notably, inflammatory hypoxia, mainly through expression of HIF genes, contributes significantly to tumor malignance and metastasis in various cancer types. In sum, above findings provide a mechanistic involvement of the IFN-induced signaling and transcription programming with cancer development and metastasis that operate cooperatively with interactions with extracellular constituents of TME. With progressive release of pathological inflammatory cytokines and growth-induced tumor hypoxia, the transformed and infiltrated inflammatory cells of TME facilitate tumor growth and metastasis [10, 18, 46, 51]. It is therefore reasonable that IFN might, through activation of these above pathways, play a critical role in hypoxia and tumorigenesis.

Previously, our group has demonstrated a novel ISGylation of HIF-1α (a form of posttranslational modification), which leads to a negative feedback loop of hypoxic response during inflammatory IFN stimulation [36]. Here, we presented experimental data supporting that IFN-α promotes tumorigenic propensities through up-regulation of HIF-1α functions: (i) IFN-α induced expression of HIF-1α at transcriptional level; (ii) IFN-α activated the JAK/PI3K/PTEN/mTOR/AKT and Ras/p38/MEK/ERK signaling pathways to induce HIF-1α expression; (iii) HIF-1α-mediated expression of EMT genes and elevated wound-healing, invasion, EMT and anti-apoptotic abilities were observed upon IFN-α exposure; and (iv) correspondingly, pharmacological modulations of JAK, PI3K and MAPK-p38 significantly reduced the IFN-α-promoted tumorigenic and metastatic propensities. Thus, our results link the IFN-α-mediated inflammatory response to the HIF-1α expression and to tumorigenic and metastasis progression, providing evidence for a novel metastasis-promoting mechanism of cancer when situates in inflammatory hypoxia microenvironments. Pharmacological targeting of cancer cells on the reliance of supporting TMEs would seem a promising therapeutic avenue and our study might present such an example.

## Methods

### Chemicals, plasmids, antibodies, cell lines and small interference siRNA

All chemicals and antibodies unless described otherwise were from Sigma and Cell signaling, respectively. Manumycin A and rapamycin were from ENZO, FH535 and JAK inhibitor I were from Calbiochem, while SP600125 and 2-methoxy-estrodiol were from Cayman. AKT inhibitor IV, IFN-α-2a and IFN-γ were from Santa Cruz, Roche and Peprotech, respectively.17-AAG was from Medchem Express. Plasmids pshHIF-1α, pXP2-Twist-HRE and pRL-tk were from Dr. KJ Wu (CMU, Taiwan), and both pHA-GSK3β-WT and pHA-GSK3β-S9A plasmids were kindly provided by Dr. J. Sadoshima (PSU, USA). GST-Raf plasmid was a kind gift from Dr. TL Shen (NTU, Taiwan). IkBα-M mutant was a gift from MR Chen (NTU, Taiwan). The plasmids including pHA-HIF1α-DM and pBabe-HA-VHL were obtained from Addgene. Antibodies against fibronectin, vimentin, actin (Sigma), HIF-1α, HIF-1β, E-cadherin, N-cadherin, β-catenin, Bcl-2, MCL-1 (BD Bioscience), STAT1_Y701_ (Invitrogen), CA9, Glut1, PGK1, 4EBP1, MDR1, S6K, S6 and mTOR, Survivin (Genetex), IFN-alpha antibody (R&D systems, MN, USA), Bmi1 (Millipore Inc.) were obtained commercially. STAT1 antibody was from Dr. CK Lee (NTU, Taiwan). Human 769-P, Caki-1 renal carcinoma, MDA-MB-231, MDA-MB-453, MCF7 breast cancer, SKOV3 ovarian cancer, SW480, DLD-1, LoVo, COLO205, HT29, RKO, Ls 174 T colorectal cancer cell lines were from ATCC and cultivated in DMEM media (except Ls174T in MEM media) with 10% fetal bovine serum (FBS), 2 mM glutamine, 100 units/ml penicillin and 100 μg/ml streptomycin in a 37 °C incubator. Simulated hypoxia was achieved by either exposure to 260 μM desferoxamine or placing cells in a hypoxia incubator (1% O2, 5% CO_2_ and 94% N2, ASTEC). Ambion STAT1 (s278), RELA/NF-κB (s11914, Life Technologies) siRNAs and ON-TARGETplus SMARTpool siRNAs targeting Akt or mTOR (GE Dharmacon) were purchased commercially.

### RNAi knockdown, transfection and immunoblotting assay

RNAi-mediated knockdown of gene expression was achieved by either transfection of siRNAs or pshHIF-1α plasmid. Transfection of siRNAs (0.1–0.3 μM) and plasmids were conducted respectively using Lipofectamine RNAiMAX (Invitrogen) and Lipofectamine 2000 (Life Technology) with the manufacturer’s protocols. After 48 h, cells were harvested and lysed for immunoblotting assay as described [36].

### Reverse transcription-polymerase chain reaction, luciferase reporter and colonogenic assays

RT-PCR and luciferase reporter assay were employed to determine gene expression. Trizol-isolated RNA were reversely transcribed by random primers and SuperScript III reverse transcriptase (Invitrogen) and cDNA pools were amplified with following primers (MB Mission Biotech): 5-‘ATGGATCCAATGGTTCTCACAGATGAT-3’ and 5’-ATGATATCTTATACGTGA ATGTGGCCTGT-3′ for HIF1α; 5’-TCCACCGCTAT GGGGAG AGCA-3′ and 5’-AGCTCCAGGATGGTGACCTTC-3′ for Glut1; 5′-ATG TCCACCAGGTCCGTG -3′ and 5’-GGATTTCCTCTTCGTGGAGT-3′ for Vimentin; 5’-ATGCCGCGCTCCTTCCTGGTC-3′ and 5’-TAATGTGTCCTTGAAGCAACCA GG-3′ for Slug; 5’-TCATGGCCAACGTGCGGGAGC-3′ and 5’-CCAGACCGAGA AGGCGTAGCT-3′ for Twist;; 5’-AGCTACTGCCATCCAATCGC- 3′ and 5’-GGGC GAATCCAATTCCAAGAG-3′ for VEGF-A; 5’-GCTGGAAGGTGGACAGCGAG- 3’and 5’-TGGCATCGTGATGGACTCCG-3′ for β-actin as a normalization control. PCR products were separated on 2% agarose gel, stained and visualized with a Dolphin-Doc system (Wealtec). Both luciferase reporter and clonogenic assays were done as described [36].

### Wound-healing and invasion assays

Wound-healing and invasion assays were used to determine cellular migratory and invasion abilities of cells. Cells (~ 80% confluent) were exposed with or without inhibitors for 30 min and treated with IFN-α for another 24 h. For wound-healing assay, cells were scrapped smoothly to generate gap and images were captured. For invasion assay, 1.25 × 10^5^ cells were seeded into Matrigel-coated transwell inserts (8-μm pore size) with different treatments in serum-free medium, and 10% FBS media in lower chamber acting as chemoattractant. After 48 h, cells those did not invade were wiped out with cotton swab and those invaded into the underside of membranes were fixed in methanol, stained with 0.5% crystal violent and scored under a microscope (100X objective). Six fields of each sample were captured to evaluate the average number of invaded cells.

### GST-Raf pull-down assay for activated Ras

GST-Sepharose beads (20 μl, GE Healthcare) was incubated with bacterial lysates with GST or GST-Raf fusion proteins in a final volume of 300 μl of buffer A (1 mM DTT, 20% glycerol, 50 mM Na_2_H_2_PO_4_ pH 8.0, 300 mM NaCl and 1 mM PMSF) at 4 °C for an hour with rotation, washed 5 times with 0.8 ml of buffer A with 1% Triton X-100 and once with 0.8 ml of buffer B (10 mM Tris pH 7.5, 1 mM EDTA, 210 mM NaCl, 15% glycerol, 0.25% NP-40, 1 mM DTT, 1 mM Na_3_VO_4_, 1 mM PMSF and 1X protease inhibitor cocktail. Beads were then reacted with a 300 μl mixture containing cell lysates, 1X protease inhibitor cocktail, 1 mM DTT and 1 mM PMSF at 4 °C for hour with rotation, washed 3 times with buffer B, boiled for 7 min in 1X SDS sample buffer, and then subjected to immunoblotting analysis.

### Extraction of cytoplasmic and nuclear fraction protein

Fractionation of 769P cells was performed using NE-PER nuclear and cytoplasmic extraction reagents according to manufacturer protocol (Thermo Scientific). The protein contents of cytoplasmic (supernatant) and nuclear extracts were determined by electrophoresis and immunoblotting analyses.

### The vasculogenic mimicry (VM) assay

The VM assay was performed with matrigel (Corning) following the manufacturer’s protocol. Briefly, Matrigel was thawed at least for two hours at 4 °C. 100 μl of the de-thawed matrigel was used to coat the wells of a 96 well plate and was allowed to polymerize for 2 h at 37 °C. After equilibrating the gel with the complete growth medium, 4 × 10^4^ cells were seeded in each well and incubated for 24 h. The tube formation was observed under a phase contrast microscope.

### The anchorage-independent cell growth/ soft- agar assay

For the soft agar assay, cells (2 × 10^4^) exposed to pharmacology inhibitors or not were mixed with 2 mL of 0.3% agarose-low EEO (Bionovas)-containing DMEM and then overlaid onto a 2 mL layer of pre-coated 0.6% agarose-containing DMEM in 6-well plates. After cultivation for 3–4 weeks, colonies were stained using 0.005% crystal violet and the number of colonies was scored on 6 random fields triplicate under microscope in three independent experiments.

### Sphere-formation and xenograft tumor growth assays

Here, sphere-formation and xenograft tumor assays were employed to determine the tumorigenic activities of human 769P renal cancer cells with IFN-α treatment and/or HIF-1α knockdown in vitro and on nude mice in vivo [36, 52]. For the sphere-formation assay, cells were transfected with pSuper vector control or pshHIF-1α plasmid for one day, followed by addition of IFN-α (1000 U/ml, 24 h) and then cells were counted and plated at a cell density of 1000 cells per well in ultra-low attachment 24-well plate (Corning) containing 1 ml of serum-free DMEM/F12 supplemented with 2% B27 (Invitrogen), human recombinant fibroblast growth factor 2 (FGF-2, 20 ng/ml) and epidermal growth factor (EGF, 20 ng/ml, Peprotech). Severn days after plating, total sphere colonies formed in each well were counted under microscope. The protocol for animal experiments was designed in accordance with the guidelines of the Institutional Animal Care and Use Committee and approved by the same (No. 20160242). For the tumorigenic xenograft model, 4- to-5-week-old BALB/c nude mice were supplied by Bio LASCO and the experiment was carried out in the Animal Center at National Taiwan University College of Medicine (NTUCM) and maintained in the specific pathogen-free (SPF) facility. A total of 10^7^ cells were re-suspended in 100 μl mixture containing 50 μl of 1X PBS and 50 μl of matrigel, and then injected subcutaneously into the dorsal region of the nude mice. Tumor size was measured every 2-to-4 days using calipers and tumor volumes were estimated by the following formula: V = ab^2^/2 (a, length; b, width).

### Quantitative measurements and statistical analyses

Quantitative measurements of obtained results were performed using ImageQuant program (Molecular Dynamics) and presented as the mean ± standard error of mean (S.E.M.) of at least three independent experiments (*n* ≥ 3). Statistical analyses were performed using the simple Student’s *t*-test. Data were considered to be significant if the *P* value was less than 0.05 (*P* < 0.05).

## Results

### Tumor heterogeneity existed in the interferon (IFN)-induced up-regulation of functional HIF-1α

**Fig. 1:**
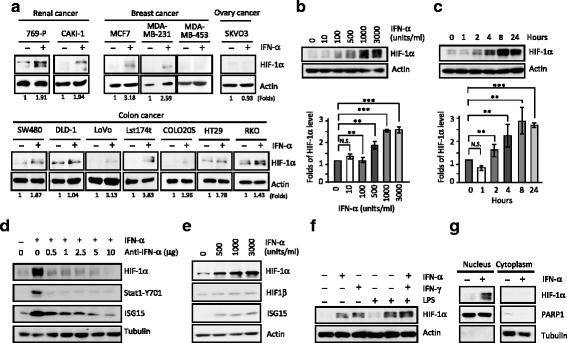
Interferon-α-induced up-regulation of HIF-1α expression in cancer cells is heterogeneous, but concentration- and time-dependent. **a** Most of cancer cell lines responded to interferon-α (IFN-α) with higher HIF-1α protein expression. **b-c** IFN-α induced HIF-1α expression in 769P cells is dose- (**b**, fix 24 h, indicated dosage) and time-dependent (**c**, fixed 1000 units/ml IFN-α, indicated times). **d** IFN-α specific antibody attenuated IFN-α induced HIF-1α expression, (**e**) 769P cells treated with IFN-α for 24 h had a dose-dependent (500, 1000 and 3000 units/ml) elevated expression of HIF-1α and ISG15, but not that of HIF-1β. **f** IFN-α (1000 units/ml) and IFN-γ (100 ng/ml), but not lipopolysaccharide (LPS, 10 μg/ml), greatly elevated HIF-1α expression. **g** IFN-α induced HIF-1α expression was majorly on the nucleus fractions, but not cytoplasm

Hypoxia and inflammatory responses play critical roles in TME and their dysfunction often link to tumorigenesis. Our previous study has shown a novel negative regulatory loop of HIF-1α activity by induction of IFN-stimulated gene 15 (ISG15) and ISGylation [36]. Here, we further examined potential effects of IFN-α on HIF-1α expression in a panel of renal, breast, ovary and colorectal cancer cell lines (Fig. 1a). Heterogeneous basal levels of HIF-1α were observed, but notably, IFN-α stimulated increased expression of HIF-1α in ~ 84.6% (11/13) of cell lines examined. Based on the above results, we thus chose the most prominent 769-P cells as a model system for following studies and observed a dose- and time-dependent IFN-α increased HIF-1α expression in 769-P cells (Fig. 1b and c; quantitative data, *lower* panels). Notably, this response is directly mediated through the IFN-α signaling as evidenced by the observation that antibodies against IFN-α showed a dose-dependent blockage of the IFN-induced HIF-1α expression (Fig. 1d). Moreover, HIF-1β expression level was only minimally affected by IFN-α treatment (Fig. 1e) and only IFN-α and IFN-γ but not lipopolysaccharide (LPS), stimulated HIF-1α expression (Fig. 1f). Furthermore, IFN-α induced an increased HIF-1α expression was majorly in the nucleus fraction, indicating its functional role IFN inflammatory response (Fig. 1g).

**Fig. 2:**
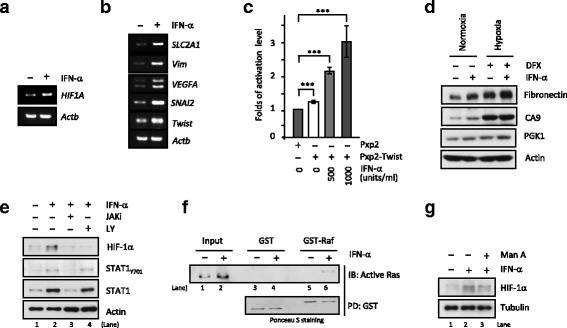
Functional HIF-1α expression induced by interferon-α depends on active transcription. **a** Analysis of *HIF-1α* mRNA level revealed a transcription dependence of IFN-α-induced HIF-1α expression. **b-d** IFN-α-increased HIF-1α proteins are functional as evidenced by RT-PCR analysis of elevated mRNA levels of HIF-1α-targeting genes (**b**), luciferase reporter assay with Twist promoter upon treatment with IFN-α (500 and 1000 units/ml) (**c**) and immunoblotting analysis of higher protein expressed of HIF-1α target genes CA9 and PGK1 under normoxia and hypoxia (**d**). Cells were treated with 1000 units/ml IFN-α for 24 h (unless indicated otherwise) before lysis and subsequent analyses. **, *P* < 0.01; ***, *P* < 0.001 (**e**) IFN-α treatment activated JAK pathway as shown by phosphorylation of STAT1_Y701_ and both JAK (JAKi, 10 μM) and PI3K inhibitors (LY294002, LY, 10 μM) abrogated HIF-1α expression. **f** IFN-α activated Ras pathway. IFN-α-treated cell lysates (24 h) were subjected to Raf pull-down assay for the level of activated Ras. **g** Ras farnesyltransferase inhibitor Manumycin A (Man A, 10 μM) inhibited HIF-1α expression

Next, we sought to examine mechanism(s) underlying this IFN-α-induced HIF-1α expression and whether functional HIF-1α pathway is activated by IFN-α? RT-PCR results revealed that IFN-α induces *HIF-1α* mRNA expression (Fig. 2a) and DRB transcription inhibitor blocks the IFN-α-induced HIF-1α expression (Additional file 1: Figure S1A). HIF-1α is a master hypoxia transcription factor mediating expression of a defined set of genes with different physiological and pathological functions, such as epithelial–mesenchymal transition (EMT), angiogenesis and metastasis (i.e. termed hypoxic responses). In Fig. 2b, mRNA levels of HIF-1α-targeted genes *SLC2A1*, *Vim*, *VEGFA*, *SNAI2* and *Twist* were induced by IFN-α treatment suggesting that elevated HIF-1α proteins in IFN inflammatory response are functional. Consistently, we have found that IFN-α induced HIF-1α transcriptional activity on the *Twist* promoter using a reporter assay (Fig. 2c). Moreover, IFN-α could also induce protein expression of two HIF-1α-targeting genes (CA9 and PGK1) and EMT marker fibronectin under both normoxia and desferoxamine (DFX) -induced mimetic hypoxia conditions (Fig. 2d). Furthermore, IFN-α induced HIF-1α expression in the presence of proteasome inhibitor MG-132 but IFN-α did not affect HIF-1α degradation (Additional file 1: Figure S1B-C). Consistently, IFN-α induced HIF-1α expression in the VHL-deficient 769-P cells expressing functional VHL E3 ligase (Additional file 1: Figure S1D). In addition, though HIF-1α level changes during the course of hypoxia TME (1% O_2_ for 3, 6, 9 or 12 h), IFN-α could still induced HIF-1α expression at the 6- and 9- h treatment of 1% O_2_ (Additional file 2: Figure S2).

### JAK and Ras axes contribute to HIF-1α expression induced by IFN-α

Using pharmacological and genetic approaches, we next examined which signaling pathways, including classical JAK-STAT pathways and auxiliary pathways such as MAPK-P38 and PI3K/mTOR/AKT axes, contribute to the IFN-α-induced HIF-1α expression? IFN-α treatment activated JAK kinase as evidenced by phosphorylation of STAT1 at Tyrosine 701 (Fig. 2e, STAT1_Y701_, comparing lane 1 and 2) and JAK inhibitor (JAKi) blocked both the IFN-α-induced STAT1_Y701_ and HIF-1α expression (lane 2 and 3). We also found IFN-α activated Ras pathway as that GST-Raf proteins pulled down more activated Ras from the IFN-α-treated extract (Fig. 2f,comparing lane 5 to 6) and consequently Ras farnesyltransferase inhibitor Manumycin A reduced the IFN-α-induced HIF-1α expression (Fig. 2g,comparing lane 2 to 3). Together, our results thus suggested an activated JAK and Ras axes regulates the IFN-α-induced HIF-1α expression.

**Fig. 3:**
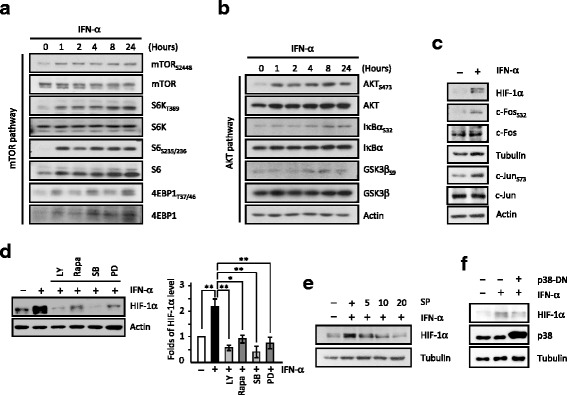
IFN-α exposure activates downstream AKT/mTOR/GSK3β, NF-κB and MAPK/JNK signaling pathways. **a-c** IFN-α treatment leads to extensive activation of the mTOR (**a**), AKT, GSK3β, IκBα (**b**) and JNK pathways (**c**). **d-e** Pharmacological inhibition of PI3K (LY, 10 μM), mTOR (rapamycin, Rapa, 0.2 μM), p38 (SB203580, SB, 20 μM), ERK (PD98059, PD, 40 μM; **d**, quantitation data, *right* panel) and JNK (SP600125, SP, 5, 10, 20 μM; **e**) all led to reduction of IFN-α-induced HIF-1α expression. **f** Functional blockage of p38 kinase in the p38-DN-expressing cells reduced HIF-1α expression

We further determined whether the downstream Raf/p38/ERK/JNK pathways and PI3K/mTOR/AKT are involved in the JAK- or Ras-directed HIF-1α expression induced by IFN-α. First, we found that IFN-α activated mTOR (including mTOR_S2448_, S6K_T389_, S6_S235/236_, and 4EBP1_T37/46_; Fig. 3a), AKT (including AKT_S473_, IκBα_S32_ and GSK3β_S9_; Fig. 3b) and p38/ERK/JNK pathways (including c-Fos_S32_ and c-Jun_S73_, Fig. 3c). Consistently, inhibitors of PI3K (LY294002, Fig. 2e), mTOR (rapamycin), p38 (SB203580), ERK (PD98059, Fig. 3d) and JNK (SP600125, Fig. 3e) all reduced the IFN-α-induced HIF-1α expression. Moreover, expression of dominant negative p38 mutant (p38-DN) also resulted in a caused a decrease in the IFN-α-induced HIF-1α expression (Fig. 3f).

### JAK with PI3K/PTEN/mTOR/AKT/GSK3β/β-catenin axis and of Ras with p38/MEK/ERK/JNK pathway regulate the IFN-α-induced HIF-1α expression

The above observation that LY294002 affected the IFN-α-induced HIF-1α, but not STAT1_Y701_ expression (Fig. 2e) indicates no role of STAT1 in the IFN-α-induced HIF-1α expression. Consistently, si-STAT1 knockdown only minimally impacted the IFN-α-induced HIF-1α expression in normoxia and hypoxia-mimetic desferoxamine (DFX) treatment (Additional file 3: Figure S3A).

**Fig. 4:**
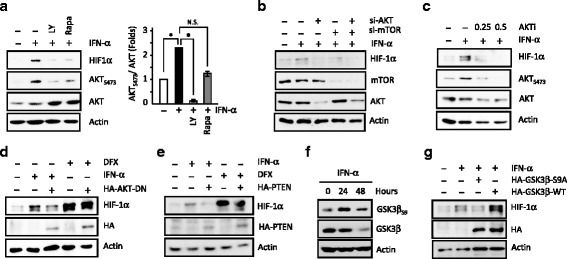
The IFN-α-induced HIF-1α expression is mediated through downstream PI3K/AKT/mTOR/GSK3β pathway. **a** Both the activation of AKT_S473_ and HIF-1α expression induced by IFN-α were blocked by co-treatment of PI3K (LY, 10 μM) or mTOR inhibitors (Rapa 0.1 μM). Quantitation data is shown in the *right*. **b-e** The IFN-α-induced HIF-1α expression is PI3K/AKT/mTOR-dependent. HIF-α expression is abrogated in si-AKT and si-mTOR knockdown cells (**b**), by exposure to AKT inhibitor iv (AKTi, **c**), in the AKT-DN- (**d**) and PTEN-expressing cells (**e**). **f** IFN-α treatment activates time-dependent GSK3β_S9_ phosphorylation and degradation. **g** Phosphorylation of GSK3β_S9_ is involved in the IFN-α induced HIF-1α expression. Inhibitors were pretreated 30 min before exposure to IFN-α and subsequently corresponding experiments were carried out as described in “Methods”. Transient plasmid transfections were performed as described 24-h before treatments. *, *P* < 0.05; N.S., non- significant

In Fig. 4a, we found that the IFN-α-induced AKT_S473_ and HIF-1α expression were both reduced by LY294002 or rapamycin co-treatments (quantitative data, *right* panel) and thus suggesting a potential involvement of the PI3K/AKT/mTOR pathway. In this regard, si-AKT, si-mTOR or AKT inhibitor iv (AKTi) all reduced the IFN-α-induced HIF-1α expression (Fig. 4b-c). Consistently, expression of AKT-DN only decreased HIF-1α expression induced by IFN-α but not by DFX treatment (Fig. 4d). Moreover, ectopic expression of negative regulator PTEN also blocked both the IFN-α-induced HIF-1α (Fig. 4e), and AKT_S473_ expression (Additional file 3: Figure S3B).

Many downstream pathways, such as GSK3β and IKK/IκB/NF-κB, play important roles in the AKT-mediated cellular responses. Here, we showed that IFN-α treatment induced GSK3β_S9_ phosphorylation and subsequent GSK3β degradation (Figs. 3b and 4f and Additional file 3: Figure S3B). Ectopic expression of GSk3β-S9A mutant, but not GSK3β-WT, reduced the IFN-α-induced expression of HIF-1α (Fig. 4g). Moreover, β-catenin inhibitor FH535 also reduced the IFN-α-induced expression of HIF-1α, cyclin D1, and active β-catenin (Additional file 3: Figure S3C-D). Further, IFN-α treatment only slightly activated IKK/IκBα_S32_ pathway and subsequent IκBα degradation (Fig. 3b and Additional file 4: Figure S4A). In agreement, IKK inhibitor sulfasalazine, expression of IκBα non-degradable mutant (IκBα-M) and si-p65 only affected the IFN-α-Induced HIF-1α expression minimally (Additional file 4: Figure S4B-D).

### IFN-α treatment induces expression of EMT genes, wound-healing, invasion, and anti-apoptotic abilities where HIF-1α plays a critical role

**Fig. 5:**
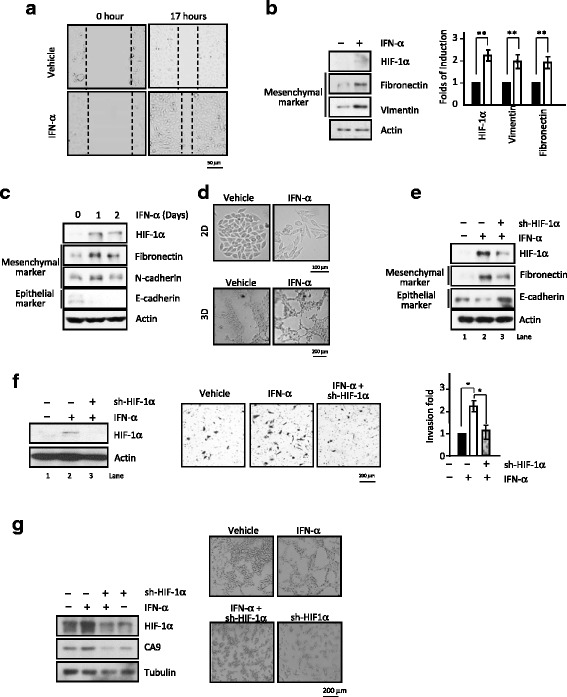
Funtional HIF-1α expression plays a critical role in IFN-α-stimulated tumorigenic propensities. **a** Wound-healing ability is increased upon IFN-α exposure. Gap images were captured under microscope at post-treatment time of 0 and 17 h. **b-d** IFN-α regulates protein expression of EMT genes, including up-regulation of mesenchymal marker proteins (**b**, quantitation data, *right* panel) and decrease in epithelia marker E-cadherin (**c**), and promotes EMT ability (**d**). The images of cellular morphology upon IFN-α stimulation were changed. In the 2D assay (the upper panel), cells were treated with or without IFN-α for 5–6 days and in the 3D assay (the lower panel), the vasculogenic mimicry of cells with or without IFN-α for 24 h. The images of both of them were observed under the phase contrast microscope. **e-g** The elevated expression of fibronectin expression, reduced E-cadherin expression (**e**), enhanced invasion activity (**f**, quantitation data, *right* panel) and increased vasculogenic mimicry (**g**; quantitation data, *right* panel) caused by the IFN-α treatment all are mediated through up-regulation of functional HIF-1α. *, *P* < 0.05 (**g**) The IFN-α-induced higher HIF-1α and downstream gene CA9 expression and knockdown of HIF-1α (left panel), totally block the expression of them and the destruction of tubule formation (right panel).The corresponding levels of HIF-1α after IFN-α treatment and/or sh-HIF-1α knockdown were determined by immunoblotting analysis and shown (**e**, *top* column; **f**-**g**, *left* panels).*,P < 0.05

**Fig. 6:**
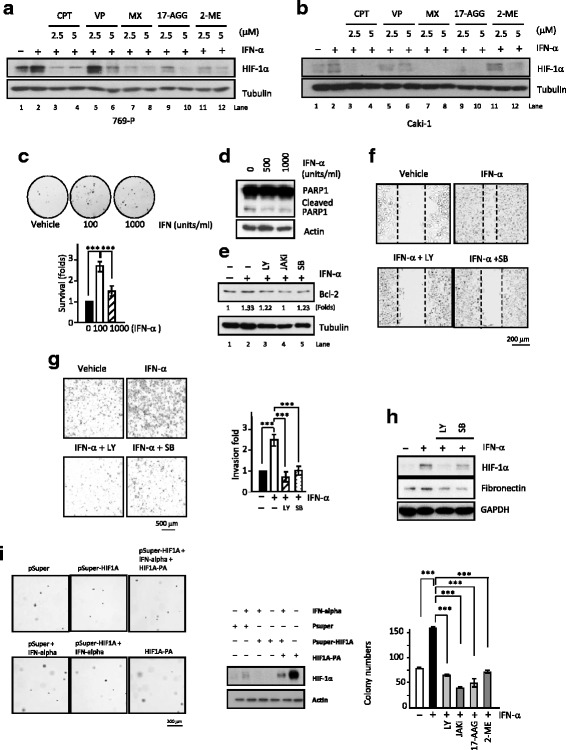
**I**nhibitors of HIF-1α and IFN-α-downstream signaling molecules also affect the levels of IFN-α-stimulated tumorigenic propensities. **a-b** Pharmacological modulating HIF-1α level by different inhibitors resulted in concentration-dependent effects on IFN-α-induced HIF1α expression in both 769-P (**a**) and Caki-1 cells (**b**). Cells were first treated with commercially available HIF-1α inhibitors, including compounds targeting Top1 (camptothecin, CPT), Top2 (etoposide, VP; mitoxantrone, MX) and HSP90 (17-N-allylamino-17-demethoxygeldanamycin, 17-AAG) as well as 2-methoxy-estradiol (2-ME), and then subjected to Western blotting analysis. **c-e**, Cellular IFN-α exposure enhanced clonogenic survival (**c**, quantitation data at the *bottom*), reduced apoptotic PARP1 cleavage (**d**) and increased Bcl-2 expression (**e**). (**f-h**) PI3K (by LY), p38 (by SB) inhibition or JAKi caused great reductions in the IFN-α-promoted wound-healing (**f**), invasion (**g**, quantitation data, *right* panel) and EMT activities (**h**) of 769-P cells. In most of the experiments, cells were exposed to 1000 units/ml IFN-α for 24 h before lysis and subsequent analyses. **i** The IFN-α-stimulated anchorage growth ability is positively related to the expression of HIF-1α (The soft-agar data, *left* panel; immunoblotting data, *middle* panel; quantitation data, *right* panel). The reduction and higher expression of HIF-1α levels are achieved by introducing sh-HIF-1α and HIF1α -PA plasmids respectively into 769-P cells. *, *P* < 0.005;** *P* < 0.01;*** *P* < 0.001

Our and other studies have shown that elevated expression of functional HIF-1α leads to elevated expression of EMT genes (Fig. 2a), wound-healing and invasion abilities. Here, we found IFN-α could effectively increase wound-healing abilities (Fig. 5a), expression of mesenchymal marker proteins (fibronectin, vimentin and N-cadherin), but decrease expression of epithelial protein E-cadherin (Fig. 5b-c) thus leading to conversion of epithelial into mesenchymal cells (Fig. 5d, upper panel) and promote tubule formation (Fig. 5d, lower panel). We therefore examined whether above IFN-α-induced biological effects are mediated through HIF-1α. In Fig. 5e, sh-HIF-1α successfully knocked down HIF-1α expression (comparing lane 2 to 3) as well as reversed the IFN-α-induced increase in fibronectin level and decrease in E-cadherin level. In Fig. 5f, IFN-α treatment increased the invasion ability, which is likely mediated by HIF-1α since sh-HIF-1α (*left* panel) reduced the IFN-α-induced invasion ability (*middle* panel and quantitative data, *right* panel). In addition, IFN-α also induced vasculogenic formation and sh-HIF-1α knockdown destruction of vasculogenic formation (Fig. 5g, right panel) and the decreased HIF-1α downstream gene CA9 expression (Fig. 5g, left panel). Inhibitors of HIF-1α, camptothecin (CPT; targeting topoisomerase I, Top1), etoposide and mitoxantrone (VP and MX, targeting Top2), Tanespimycin (17-N-allylamino-17-de- methoxygeldanamycin (17-AAG, targeting HSP90) and 2-methoxy-estradiol (2-ME), all showed concentration dependent inhibitory effects on the IFN-α-induced HIF-1α expression in both 769-P (Fig. 6a) and Caki-1 cells (Fig. 6b).

Notably, we also found that IFN-α promoted cancer cell survival as evidenced by higher colony number of IFN-α-treated cells in a clonogenic survival assay (Fig. 6c; quantitative results, *lower* panel) and reduced apoptotic cleavage of PARP-1 (Fig. 6d). These might be related to our observation that IFN-α increases Bcl-2 expression (Fig. 6e, comparing lane 1 to 2). Consistently, IFN-α co-treatment also reduced apoptotic PARP1 cleavage caused by MX (Additional file 5: Figure S5A).

### PI3K and MAPK inhibitors modulate IFN-α-promoted apoptotic, wound-healing and invasion abilities

In Fig. 6e, IFN-α-induced elevated Bcl-2 expression (i.e. possibly anti-apoptotic activity) was reduced by co-treatments of LY294002 (comparing lane 2 to 3), JAKi (comparing lane 2 to 4) or SB203580 (comparing lane 2 to 5). Thus, we investigated whether these pharmacological inhibitors have a medical intervention in preventing above IFN-α-induced tumorigenic propensities in inflammatory microenvironment? Our results revealed that LY294002 and SB203580 could both effectively inhibit not only IFN-α-induced wound-healing (Fig. 6f) but also invasion abilities (Fig. 6g, *left* panel; *middle* panel,quantitative data; Additional file 5: Figure S5B, larger images). Notably, pharmacological effects of LY294002 and SB203580 were correlated with their abilities to inhibit HIF-1α and downstream fibronectin protein expression (*right* panel). Armed together, our results clearly showed that pharmacological inhibitors could inhibit the IFN-α-induced, HIF-1α-mediated pro-tumorigenic propensities during inflammatory TME and thus maybe of some therapeutic intervention.

### Functional HIF-1α is required for IFN-induced tumorigenesis, invasion, and vasculogenic mimicry

**Fig. 7:**
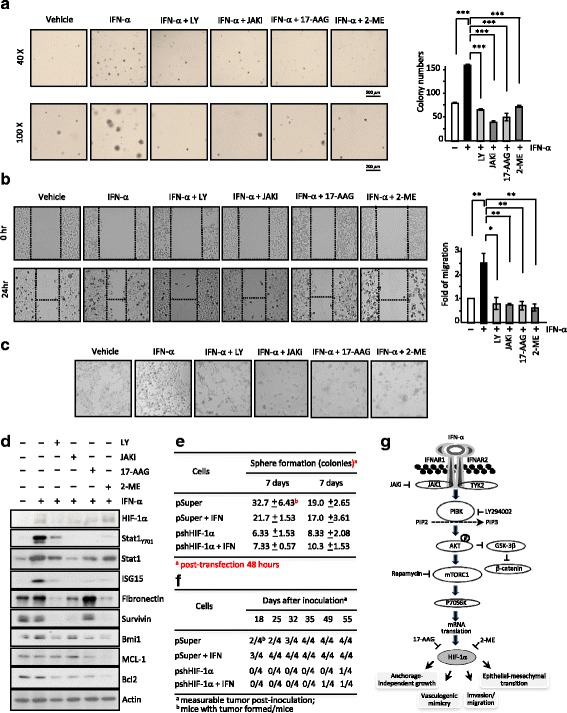
The PI3K/AKT/mTOR signaling axis and HIF-1α play roles in cellular growth, invasion, vasculogenic mimicry, sphere formation activities in vitro and tumor growth in vivo induced by acute IFN-α exposure. **a-c** Pharmacological inhibitions of the JAK/PI3K/PTEN/mTOR/AKT, Ras/p38/MEK/ERK axes and HIF-1α significantly impacted on the IFN-α-stimulated anchorage-independent growth (**a**), scratch wound closure (**b**) and vasculogenic mimicry formation (**c**) in 769-P cells. **d** Inhibitors (as indicated) differentially affected expression of genes involved in EMT, cell survival and apoptotic cell death. **e-f** HIF-1α is needed for sphere colony formation (**e**), tumor formation and growth (**f**) of differentially educated 769-P cells; i.e. pSuper, pSuper + IFN, pshHIF-1α and pshHIF-1α + IFN cells. **g** Schematic illustration of the signaling pathways involved in the IFN-α-induced HIF-1α expression and stimulated tumorigenic propensities. The IFN-α-induced H IF-1α expression by first binding to the interferon alpha receptor 1 and 2 (IFNAR1/2), subsequent activation of JAK1 and TYK2, phosphorylation of PI3K, AKT and mTOR, those lead to promotion of *HIF-1*α mRNA transcription and translation as well as corresponding tumorigenic activities including EMT, anchorage-independent growth, invasion and vasculogenic mimicry activities. **, *P* < 0.01; ***, *P* < 0.001

Since the IFN-α-induced HIF-1α expression plays a potential role in inflammatory tumor-environment, we next sought to determine possible tumor propensities offered by IFN-α in TME. Anchorage-independent growth of colony formation was carried out to examine the effect of IFN-α on tumorigenesis. As shown in Fig. 6i, IFN-α did promote colony growth on this-soft agar assay. This tumorigenic growth is dependent on HIF-1α, since when endogenous HIF-1α was knocked down by plasmid-mediated shHIF-1α expression (pshHIF-1α), the IFN-α-induced colony formation was reduced significantly (Fig. 6i: *left* panel, quantitative data). Moreover, the tumorigenic growth was also partially compensated by expressing degradation-defected HIF-1α-PA. As a positive control, HIF-1α-PA expression alone led to an increased colony formation similar to that by IFN-α. To further support the notion that HIF-1α plays a pivotal role in the IFN-α-mediated biological effects, we then examined a series of HIF-1α functional inhibitors in inhibiting the IFN-α-mediated biological functions. In Fig. 7a, LY297002, JAKi inhibitor, 17-AAG or 2-ME all diminished the tumorigenic growth on soft-agar (Fig. 7a; right panel, quantitative data), migration ability (Fig. 7b; right panel, quantitative data) and tubule formation of vasculogenic mimicry (VM, Fig. 7c) stimulated by IFN-α treatment. Consistently, the IFN-α-stimulated expression of the HIF-1α downstream genes including fibronectin, survivin, Bmi1, MCL1 and Bcl2 were also abolished with co-treatments of above inhibitors, suggesting the key role of HIF-1α in the IFN-α-induced phenomena in inflammatory-hypoxia TME (Fig. 7d). Lastly, we showed that anti-a-specific antibody also effectively antagonize the IFN-α-induced changes of biomarker expression of EMT (vimentin; Additional file 6: Figure S6A) and stemness (Bmi1; Additional file 6; Figure S6B).

As shown above, HIF-1α is required for IFN-α induced tumorigenic propensities by determining colony-formation, invasion and vasculogenic mimicry abilities in vitro with cell culture. To better understand the IFN-stimulated processes and to support the above notion that HIF-1α is required for tumorigenic propensities of IFN-treated cells, we carried out sphere-formation assay by growing differently treated cells in secondary (self-renewal) sphere culture and in vivo studies using xenograft tumor model. The HIF-1α-deficient cell lines formed sphere colonies significantly less frequently than pSuper vector-transfected cells in despite of with or without acute exposure of IFN (Fig. 7e, compared pSuper to pshHIF-1α). In addition, the in vivo tumor formation rate and tumor growth are also much lower in the psh-HIF-1α cells (Fig. 7f). It is also interesting to note that the initial time of measurable tumors of IFN-treated cells after inoculation is earlier than those of cells without acute exposure of IFN despite of HIF-1α expression status (e.g. three out of four mice with pSuper + IFN cells and two out of four mice with pSuper cells are with measurable tumor formed at 18th day after inoculation).

## Discussion

Tumor microenvironment (TME) provides supportive niche for tumor progression and establishes communication links with cancer cell survival, stemness-like property, hypoxia, inflammation and progression immunity. It has also been reported as an important step in the metastatic cascade of epithelial tumors and thus suggesting that TME could be constituted with corresponding signaling pathways served as promising target(s) for cancer therapy [53–55]. Despite of great advances in health care programs, basic, translational and clinical medical research in the past decades, cancer remains a daunting threat around the world despite great advances in health care programs, basic, translational and clinical medical research in the past decades [12, 56]. A global unmet need for understanding fundamental bases of cancer biology and new interventions of anticancer therapeutics is urgently needed [56, 57]. It has become notably apparent that generating optimal oncogenic responses needs proper signaling crosstalk between inflammatory and hypoxic pathways of TME [58]. In this regard, solid tumors often situate in hypoxic inflammatory niche containing leukocyte infiltrates in which ~ 20% of them arising in association with chronic inflammation [1–3, 7, 17, 26]. Moreover, inflammatory and hypoxic niches as well as corresponding signaling pathways play a complex role in cancer development through regulating expression of tumorigenic propensities. Thus, targeting the inflammatory interferon-driven and hypoxia-induced pathological TME and corresponding signaling pathways of our study might present as such an example for novel anticancer intervention, particularly identified signaling pathways those regulate the expression of HIF-1α in malignant tumor.

We showed that cellular signaling responses of hypoxic and inflammatory TMEs interact and crosstalk through up-regulation of expression of HIF-1α and associated tumorigenic activities, which are mediated by one inflammatory cytokine IFN-α. This novel communication of above two TMEs, via the JAK pathway with PI3K/PTEN/ mTOR/AKT/GSK3β/β-catenin axis and the Ras pathway with p38/MEK/ERK/JNK axis, further fine-tunes cellular homeostasis of tumor cells, i.e., adapting to hypoxia, enhancing survival as well as promoting migration, invasion and EMT. Our study has thereby not only revealed a new crosstalk between two key inflammatory and hypoxic TMEs, but provided a vicious molecular connection between hypoxic inflammation and tumorigenic programming. In this same line, we have recently reported a new negative feedback loop for the HIF-1α-mediated pathway involving the regulation of HIF-1α by ISG15 and ISGylation of HIF-1α [36]. Using genetic and pharmacological inhibition, our study has provided new insights into an important involvement of hypoxic inflammatory TME in tumorigenesis. In addition, clinical and/or preclinical inhibitors of signaling pathways involved in regulating HIF-1α expression used in this study also present as new strategies for treatment of human cancer. Consistently, the anti-inflammatory drugs, such as NSAID, have been reported to reduce the risk of several solid tumors [59].

Our advanced understanding of a cancer-TME interaction in molecular regulation of tumorigenesis shall lead to a rationale guidance for the development of new cancer therapeutics targeting TME-supplemented IFN signaling and HIF-1α programming to prevent disease relapse after initial diagnosis and treatment [53, 54, 60].From the points of view on interventions for metastatic cancer, HIFs and regulatory pathways of gene expression are obvious targets of interest and targeting disseminated cancer cells, corresponding reliance/molecules and signaling responses/interactions on TMEs could also be promising revenues. Moreover, mounting experimental and clinical evidence have also suggested a central role for the signaling networks operating to promote a metastatic and/or therapeutic resistance cascade in cancer development that involves interactions of AR, TMPRSS2, HGF and c-MET with critical components of TMEs [61]. In agreement, our preclinical study on that signaling inhibitors including LY297002, JAKi inhibitor, 17-AAG and 2-ME all diminished the tumor propensities and growth also supports the novel usage of these signaling inhibitors in anti-cancer therapy.

IFN-α, which belongs to a family of “biologic response modifiers” and activates a network of signaling molecules, is FDA-approved for hairy cell leukemia, malignant melanoma, AIDS-related Kaposi’s sarcoma, follicular non-Hodgkin’s lymphoma as well as other clinical indications such as renal cell cancer and cervical cancer. The signaling pathways and molecules, such as PI3K, JAK, and HSP90, have also been suggested and developed into new anti-cancer strategies as well as for overcoming drug resistance [62–65]. For example, due to its frequent activation, the JAK/STAT axis is an attractive target for breast cancer therapy and thus clinical trials of JAKi in advanced breast cancer are ongoing [62]. Functional inhibition of HSP90 causes the degradation of its client proteins and thus subsequently providing a novel anti-cancer intervention to concomitantly disrupt multiple oncogenic signaling cascades especially in in a variety of client protein-driven tumors [64, 65]. Similarly, the PI3K/AKT/mTOR signaling pathway is commonly deregulated in human malignancy including non-small cell lung cancer (NSCLC) [63]. Thereby, this pathway is also an excellent target for many therapeutic development and its inhibitors are undergoing heavy clinical evaluation. In agreement, our results also showed that pharmacological inhibitors targeting the IFN-α signaling and pathways could not only modulate HIF-1α expression but also tumorigenic activities such as EMT and tumor invasion. Thus, our study provides a good example from molecular pharmacological modulations to modular tumor therapy. Specifically, we observed a novel regulation of hypoxia TME (mainly HIF-1α expression and functions) by inflammatory IFN-α. Importantly, IFN-α promotes tumorigenic propensities such as EMT, invasion and anti-apoptosis abilities and crosstalk to hypoxic TME mainly through up-regulation of HIF-1α expression [55, 58, 66–69]. Coupled with the importance of hypoxia and inflammation during tumorigenic processes, our reports on physical, functional and genetic interactions among key components of these two TMEs further suggest the critical roles of both the individual pathway of and the interaction between IFN-α and HIF-1α for the underlying tumorigenic mechanism(s) in the context of hypoxic inflammation. The observations that the IFN-induced ISG15 conjugation (ISGylation) pathway can modulate the cancer cell-killing activity of drugs also support the above notion [70, 71].

Tumor is a complex biomass containing heterogeneous cancer cells and TME with the surrounding stromal, infiltrated immune cells and extracellular matrices. It is known that TME plays critical yet diverse roles in various stages of cancer development, but mechanisms underlying signaling crosstalk and molecular communications of TMEs with oncogenic programs remain unclear. Through studying molecular mechanisms underlying interactions of inflammatory molecule interferon (IFN) with hypoxic TME, we provided the first experimental evidences for a novel communicating mechanisms occurring within TMEs in cancer progression of renal oncogenic development, thus representing as one emerging paradigm of cancer pathology. We unraveled a hypoxic pro-inflammatory role of IFN-α in the HIF-1α mediated TME and then leading to promotion of tumor tumorigenesis, migration/ invasion, EMT, vasculogenic mimicry and drug resistance (schematic illustration in Fig. 7e). Importantly, pharmacological modulations of HIF-1α as well as the JAK/PI3K/PTEN/mTOR/AKT and Ras/p38/MEK/ERK signaling axes all significantly reduced the above IFN-α promoted tumorigenic propensities. In this regards, advances in the understanding of cancer-TME interaction and the drugs targeting TME-associated disease biology and pathology may bring about the TME-guided therapy for various diseases.

## Conclusions

In conclusion, our study not only revealed a new crosstalk between inflammatory and hypoxic TMEs but also provide a vicious molecular connection between hypoxic inflammation TME and oncogenic development. Our findings of this cancer-TME interaction in tumorigenesis might thereby lead to a rationale guidance/strategy for developing new therapeutic interventions targeting TME-supplemented IFN signaling and HIF-1α programming (in tumor cells) to prevent disease relapse after initial diagnosis and treatment.

## Additional files

Additional file 1: **Figure S1**. IFN-α-induced HIF-1α expression in transcription- and translation-dependent, but 26S proteasome-independent. (A) RNA transcription is required for IFN-α mediated HIF-1α induction. 769-P cells were pre-treated 30 min. With or without 100 μM DRB and subsequently exposed for 24 h with either IFN-α (1000 units/ml) or desferoxamine (DFX, 260 μM), and protein labels determined by immunoblotting analysis with indicated antibodies. (B-C) Interferon-α treatment promotes the de novo synthesis of HIF-1α expression (B) and does not impact on the stability of HIF-1α. (C) Cells were treated with IFN-α before addition of (DRB, 100 μM), cycloheximide (CHX, 100 μM) or MG-132 (10 μM) (D) IFN-α stimulated HIF-1α expression similarly in the VHL-deficient 769-P cells with or without ectopic expression of functional VHL. (PPT 251 kb)

Additional file 2: **Figure S2**. IFN-α could up-regulate HIF-1α expression in the presence of 1% O_2_ with a different induction kinetics. Cells were treated with or without IFN-α, exposed to hypoxia (1% O_2_) and then harvested at indicated time points (3, 6, 9 and 12 h) for immunoblotting. (PPT 162 kb)

Additional file 3: **Figure S3**. The JAK/PI-3 K, AKT/GSK3β and p38/ERK/JNK axes contributed to the IFN-α-induced HIF-1α expression. (A) Knockdown of STAT1 (si-STAT1) expression has no effect on the IFN-α-induced HIF-1α expression. (B) Ectopic expression of PTEN antagonized the IFN-α-activated AKT/GSK3β pathway. (C-D) β-catenin inhibition by FH535 treatment not only decreased the IFN-α-induced expression of HIF-1α (C), but also reduced the IFN-α-induced active β-catenin (D). (PPT 221 kb)

Additional file 4: **Figure S4**. NF-κB is minimally involved in the IFN-α mediated HIF-1α accumulation. (A) IFN-α slightly activated IKK as suggested by a minimal increase in IkBa_S32_ phosphorylation. (B-D) Targeting IKK/IkBα/NF-κB pathway by Sulfasalazine (Sulfa, B), IkBα-M mutant (C) and si-p65 (D) do not alter much of IFN-α-induced HIF-1α expression. (PPT 201 kb)

Additional file 5: **Figure S5**. The IFN-α not only attenuated MX-induced apoptosis, but also promote PI3K- and MAPK-P38-dependent invasion activity.(A) IFN-α co-treatment reduced the MX-induced apoptotic cleavage of PARP1.(B) LY294002 (LY) and SB203580 (SB) could both effectively inhibit the IFN-α-induced invasion abilities. (PPT 237 kb)

Additional file 6: **Figure S6**. Direct effects of IFN-α on the expression of EMT and stemness biomarkers. (A-B) Cells were treated with 0.5, 1, 2.5 and 5 mg of anti-IFN-α antibodies and their impacts on the expression of EMT marker vimentin (A) and stemness marker Bmi1 genes (B) were determined by immunoblotting analysis. (PPT 133 kb)

## Abbreviations

EMT: Epithelial–mesenchymal transition
HIF-1α: hypoxia-inducible factor 1alpha
IFN: interferon
TME: Tumor-microenvironment
VM: vasculogenic mimicry
