# Supplementary material for: Inflammatory interferon activates HIF-1α-mediated epithelial-to-mesenchymal transition via PI3K/AKT/mTOR pathway
Source: J Exp Clin Cancer Res. 2018 Mar 27;37:70. doi: 10.1186/s13046-018-0730-6 (PMC5870508; doi:10.1186/s13046-018-0730-6)
Supplement: Supplementary file 1 — Figure S1. IFN-α-induced HIF-1α expression in transcription- and translation-dependent, but 26S proteasome-independent. (A) RNA transcription is required for IFN-α mediated HIF-1α induction. 769-P cells were pre-treated 30 min. With or without 100 μM DRB and subsequently exposed for 24 h with either IFN-α (1000 units/ml) or desferoxamine (DFX, 260 μM), and protein labels determined by immunoblotting analysis with indicated antibodies. (B-C) Interferon-α treatment promotes the de novo synthesis of HIF-1α expression (B) and does not impact on the stability of HIF-1α. (C) Cells were treated with IFN-α before addition of (DRB, 100 μM), cycloheximide (CHX, 100 μM) or MG-132 (10 μM) (D) IFN-α stimulated HIF-1α expression similarly in the VHL-deficient 769-P cells with or without ectopic expression of functional VHL. (PPT 251 kb) [file 13046_2018_730_MOESM1_ESM.ppt]

## Slide 1
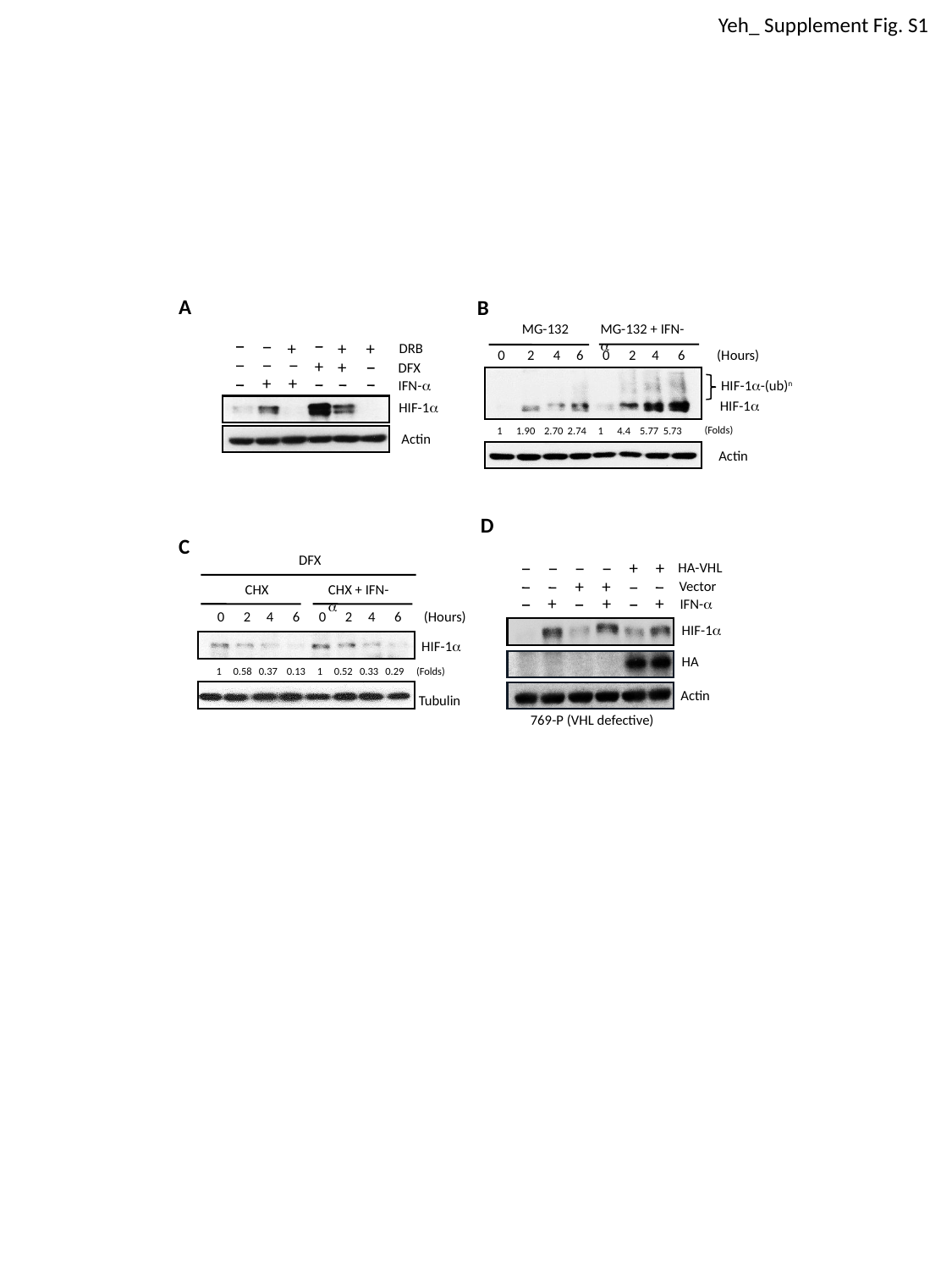

Yeh_ Supplement Fig. S1
A
B
MG-132
MG-132 + IFN-α
+
+
+
DRB
 0 2 4 6 0 2 4 6 (Hours)
+
+
DFX
+
+
HIF-1α-(ub)n
IFN-α
HIF-1α
HIF-1α
(Folds)
1 1.90 2.70 2.74 1 4.4 5.77 5.73
Actin
Actin
D
C
DFX
CHX
CHX + IFN-α
 0 2 4 6 0 2 4 6 (Hours)
HIF-1α
1 0.58 0.37 0.13 1 0.52 0.33 0.29
(Folds)
Tubulin
+
+
HA-VHL
+
+
Vector
+
+
+
IFN-α
HIF-1α
HA
Actin
769-P (VHL defective)
