# Supplementary material for: Inflammatory interferon activates HIF-1α-mediated epithelial-to-mesenchymal transition via PI3K/AKT/mTOR pathway
Source: J Exp Clin Cancer Res. 2018 Mar 27;37:70. doi: 10.1186/s13046-018-0730-6 (PMC5870508; doi:10.1186/s13046-018-0730-6)
Supplement: Supplementary file 2 — Figure S2. IFN-α could up-regulate HIF-1α expression in the presence of 1% O2 with a different induction kinetics. Cells were treated with or without IFN-α, exposed to hypoxia (1% O2) and then harvested at indicated time points (3, 6, 9 and 12 h) for immunoblotting. (PPT 162 kb) [file 13046_2018_730_MOESM2_ESM.ppt]

## Slide 1
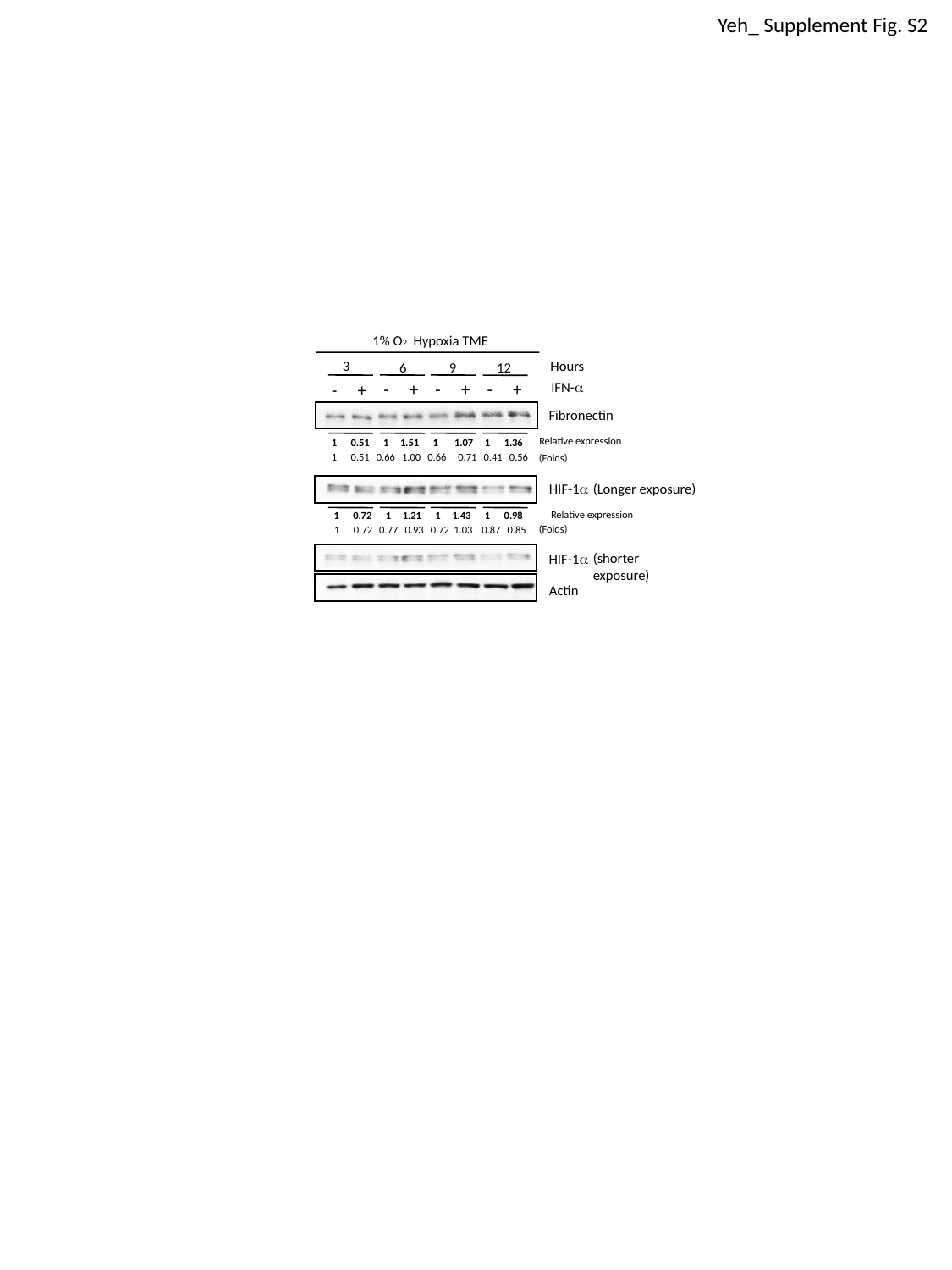

Yeh_ Supplement Fig. S2
1% O2 Hypoxia TME
3
 Hours
9
6
12
-
+
-
+
-
+
IFN-α
-
+
Fibronectin
Relative expression
 1 0.51 1 1.51 1 1.07 1 1.36
 1 0.51 0.66 1.00 0.66 0.71 0.41 0.56
(Folds)
HIF-1α
(Longer exposure)
Relative expression
 1 0.72 1 1.21 1 1.43 1 0.98
(Folds)
 1 0.72 0.77 0.93 0.72 1.03 0.87 0.85
(shorter exposure)
HIF-1α
Actin
