# Supplementary material for: Inflammatory interferon activates HIF-1α-mediated epithelial-to-mesenchymal transition via PI3K/AKT/mTOR pathway
Source: J Exp Clin Cancer Res. 2018 Mar 27;37:70. doi: 10.1186/s13046-018-0730-6 (PMC5870508; doi:10.1186/s13046-018-0730-6)
Supplement: Supplementary file 3 — Figure S3. The JAK/PI-3 K, AKT/GSK3β and p38/ERK/JNK axes contributed to the IFN-α-induced HIF-1α expression. (A) Knockdown of STAT1 (si-STAT1) expression has no effect on the IFN-α-induced HIF-1α expression. (B) Ectopic expression of PTEN antagonized the IFN-α-activated AKT/GSK3β pathway. (C-D) β-catenin inhibition by FH535 treatment not only decreased the IFN-α-induced expression of HIF-1α (C), but also reduced the IFN-α-induced active β-catenin (D). (PPT 221 kb) [file 13046_2018_730_MOESM3_ESM.ppt]

## Slide 1
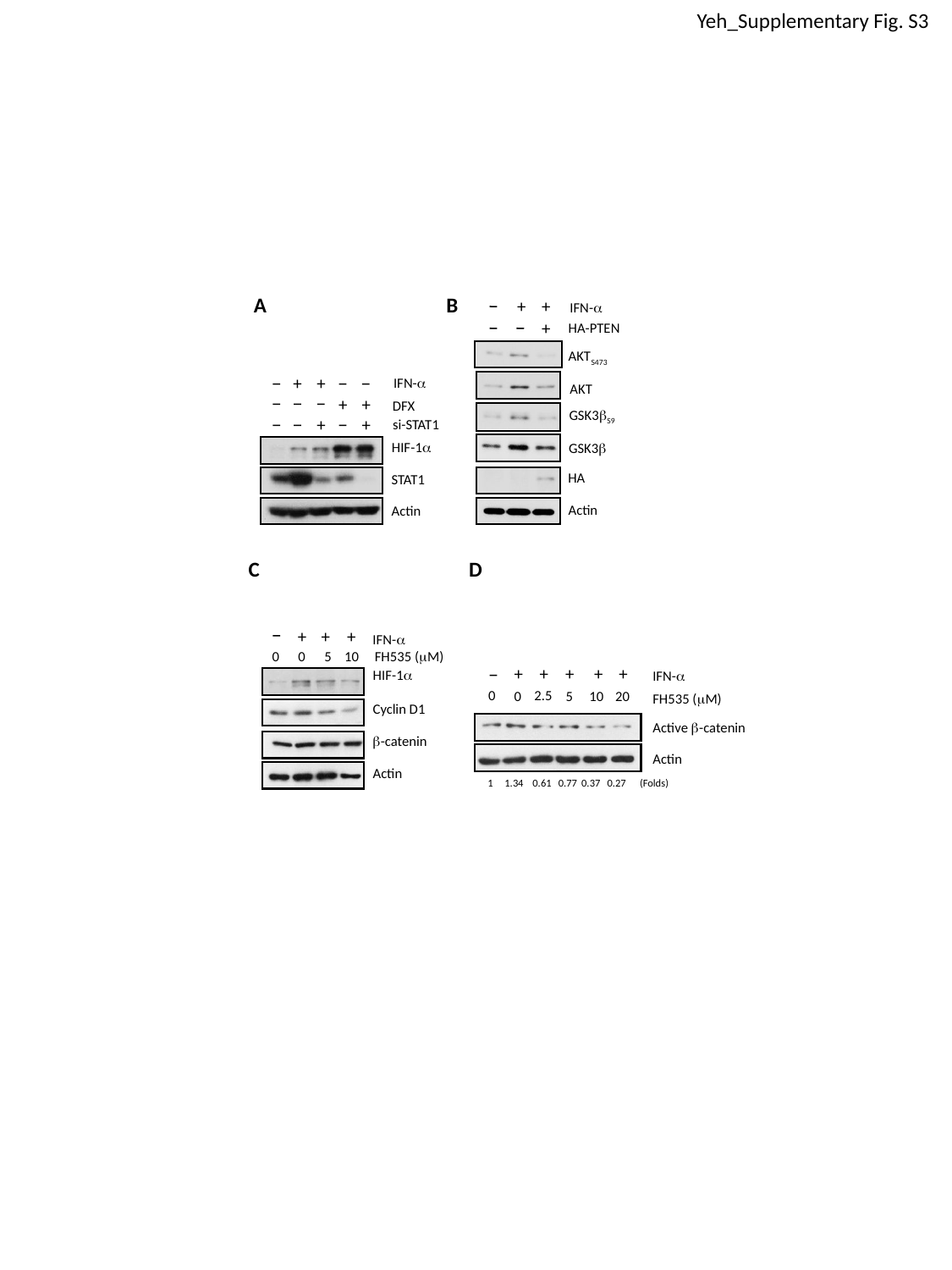

Yeh_Supplementary Fig. S3
A
B
+
+
IFN-α
+
HA-PTEN
AKTS473
+
+
IFN-α
AKT
+
+
DFX
GSK3βS9
+
+
si-STAT1
HIF-1α
GSK3β
HA
STAT1
Actin
Actin
C
D
+
+
+
IFN-α
0 0 5 10 FH535 (μM)
+
+
+
+
+
HIF-1α
IFN-α
0
2.5
0
 5
10
20
FH535 (μM)
Cyclin D1
Active β-catenin
β-catenin
Actin
Actin
1 1.34 0.61 0.77 0.37 0.27 (Folds)
