# Supplementary material for: Inflammatory interferon activates HIF-1α-mediated epithelial-to-mesenchymal transition via PI3K/AKT/mTOR pathway
Source: J Exp Clin Cancer Res. 2018 Mar 27;37:70. doi: 10.1186/s13046-018-0730-6 (PMC5870508; doi:10.1186/s13046-018-0730-6)
Supplement: Supplementary file 4 — Figure S4. NF-κB is minimally involved in the IFN-α mediated HIF-1α accumulation. (A) IFN-α slightly activated IKK as suggested by a minimal increase in IkBaS32 phosphorylation. (B-D) Targeting IKK/IkBα/NF-κB pathway by Sulfasalazine (Sulfa, B), IkBα-M mutant (C) and si-p65 (D) do not alter much of IFN-α-induced HIF-1α expression. (PPT 201 kb) [file 13046_2018_730_MOESM4_ESM.ppt]

## Slide 1
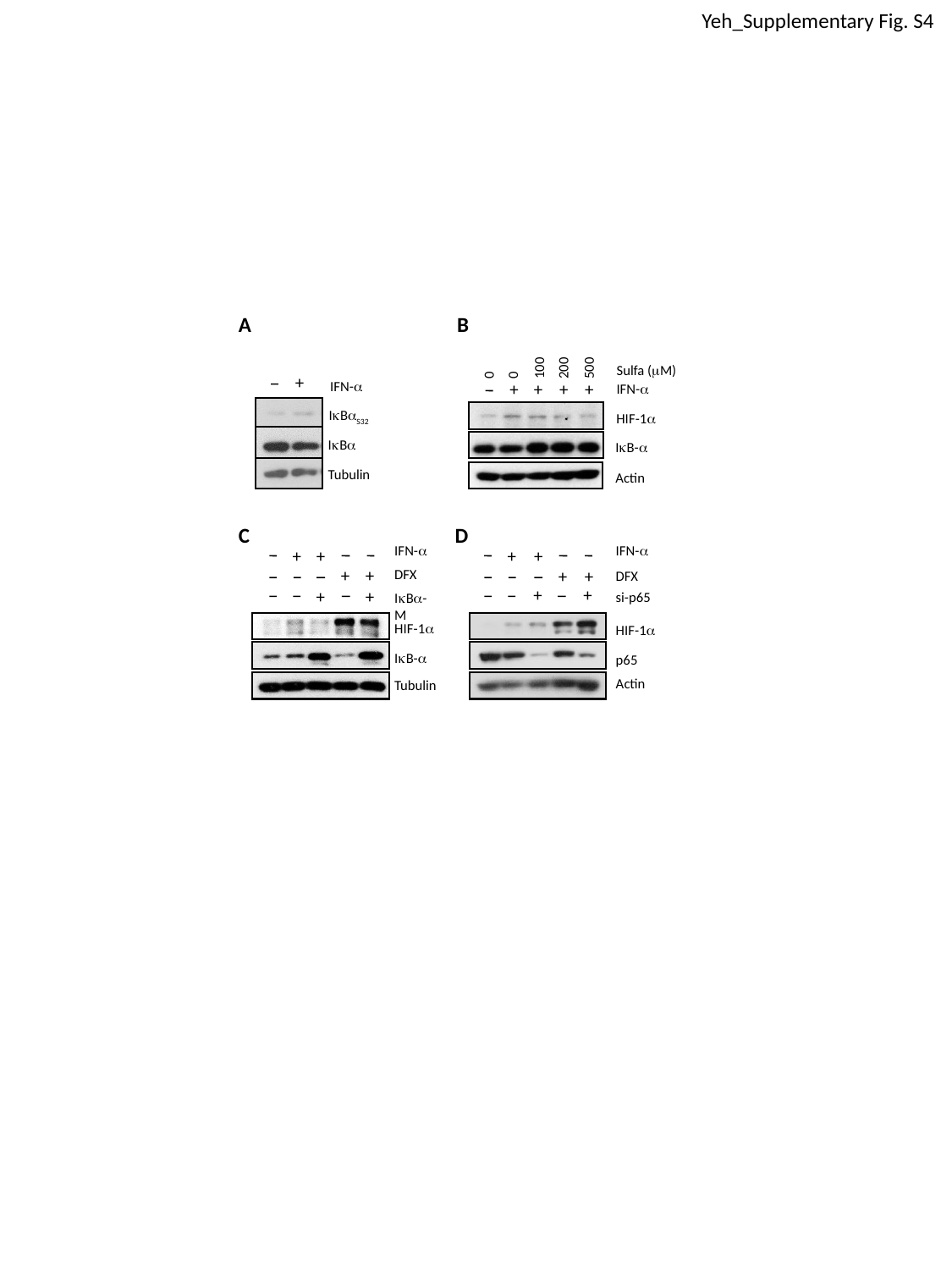

Yeh_Supplementary Fig. S4
A
B
0
100
200
500
Sulfa (μM)
0
+
IFN-α
+
+
+
+
IFN-α
IκBαS32
HIF-1α
IκBα
IκB-α
Tubulin
Actin
C
D
IFN-α
IFN-α
+
+
+
+
+
+
+
+
DFX
DFX
+
+
+
+
si-p65
IκBα-M
HIF-1α
HIF-1α
IκB-α
p65
Actin
Tubulin
