# Supplementary material for: Inflammatory interferon activates HIF-1α-mediated epithelial-to-mesenchymal transition via PI3K/AKT/mTOR pathway
Source: J Exp Clin Cancer Res. 2018 Mar 27;37:70. doi: 10.1186/s13046-018-0730-6 (PMC5870508; doi:10.1186/s13046-018-0730-6)
Supplement: Supplementary file 5 — Figure S5. The IFN-α not only attenuated MX-induced apoptosis, but also promote PI3K- and MAPK-P38-dependent invasion activity.(A) IFN-α co-treatment reduced the MX-induced apoptotic cleavage of PARP1.(B) LY294002 (LY) and SB203580 (SB) could both effectively inhibit the IFN-α-induced invasion abilities. (PPT 237 kb) [file 13046_2018_730_MOESM5_ESM.ppt]

## Slide 1
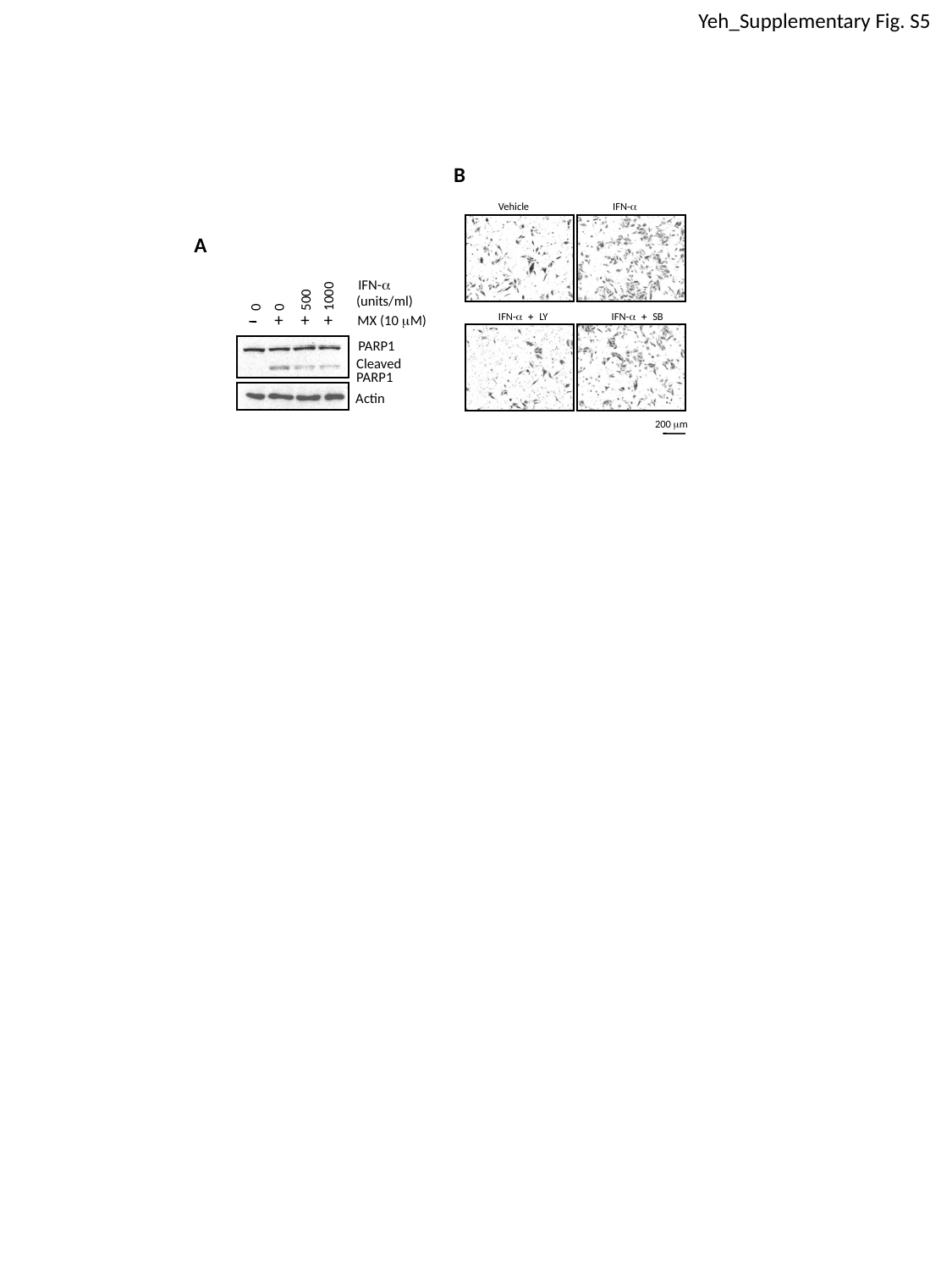

Yeh_Supplementary Fig. S5
B
Vehicle
IFN-α
A
IFN-α
500
1000
(units/ml)
0
0
+
+
+
IFN-α + LY
IFN-α + SB
MX (10 μM)
PARP1
Cleaved
PARP1
Actin
200 μm
