# Supplementary material for: Inflammatory interferon activates HIF-1α-mediated epithelial-to-mesenchymal transition via PI3K/AKT/mTOR pathway
Source: J Exp Clin Cancer Res. 2018 Mar 27;37:70. doi: 10.1186/s13046-018-0730-6 (PMC5870508; doi:10.1186/s13046-018-0730-6)
Supplement: Supplementary file 6 — Figure S6. Direct effects of IFN-α on the expression of EMT and stemness biomarkers. (A-B) Cells were treated with 0.5, 1, 2.5 and 5 mg of anti-IFN-α antibodies and their impacts on the expression of EMT marker vimentin (A) and stemness marker Bmi1 genes (B) were determined by immunoblotting analysis. (PPT 133 kb) [file 13046_2018_730_MOESM6_ESM.ppt]

## Slide 1
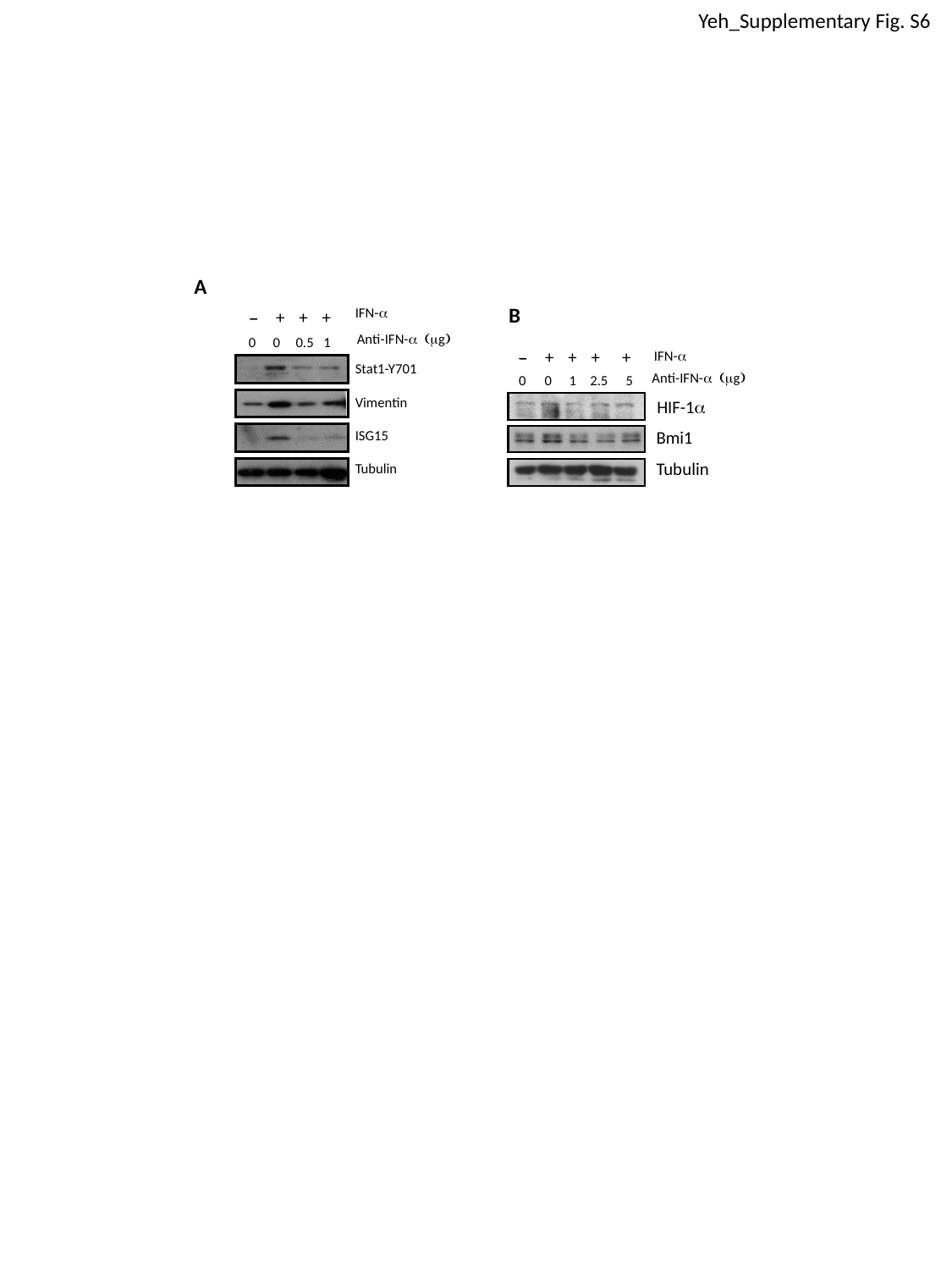

Yeh_Supplementary Fig. S6
A
B
IFN-α
+
+
+
Anti-IFN-α g
0
0
0.5
1
Stat1-Y701
Vimentin
ISG15
Tubulin
+
+
+
+
IFN-α
Anti-IFN-α g
0
0
1
2.5
5
HIF-1α
Bmi1
Tubulin
